# Diversity of sexual systems within different lineages of the genus *Silene*

**DOI:** 10.1093/aobpla/plv037

**Published:** 2015-05-15

**Authors:** Inés Casimiro-Soriguer, Maria L. Buide, Eduardo Narbona

**Affiliations:** 1 Área de Botánica, Departamento de Biología Molecular e Ingeniería Bioquímica, Universidad Pablo de Olavide, Ctra. de Utrera, km 1, 41013 Sevilla, Spain; 2 Área de Botánica, Departamento de Biología Vegetal y Ecología, Universidad de Sevilla, Avenida Reina Mercedes s/n, 41012 Sevilla, Spain

**Keywords:** *Behenantha*, Caryophyllaceae, dioecy, gynodioecy, gynodioecy–gynomonoecy, hermaphroditism, *Psammophilae*, sexual polymorphism, sexual system, *Silene*

## Abstract

Species and populations can be categorized by their sexual systems, depending on the spatial distribution of female and male reproductive structures within and among plants. Although a high diversity of sexual systems exists in *Silene*, their relative frequency at the genus and infrageneric level is unknown. Here, we carried out an extensive literature search for direct or indirect descriptions of sexual systems in *Silene* species. We found descriptions of sexual systems for 98 *Silene* species, where 63 and 35 correspond to the phylogenetically supported subgenera *Silene* and *Behenantha*, respectively. Hermaphroditism was the commonest sexual system (58.2 %), followed by dioecy (14.3 %), gynodioecy (13.3 %) and gynodioecy–gynomonoecy (i.e. hermaphroditic, female and gynomonoecious plants coexisting in the same population; 12.2 %). The presence of these sexual systems in both subgenera suggests their multiple origins. In 17 species, the description of sexual systems varied, and in most cases these differences corresponded to variations within or among populations. Interestingly, the poorly studied gynodioecy–gynomonoecy sexual system showed similar frequency to dioecy and gynodioecy in both subgenera. In addition, the incidence of gynodioecy–gynomonoecy was analysed in the species of section *Psammophilae* (*Silene littorea*, *S. psammitis*, *S. adscendens* and *S. cambessedesii*), in a survey of 26 populations across the distribution area of the species. The four species showed gynomonoecy–gynodioecy in most populations. Hermaphrodites were the most frequent morph, with a low number of females and gynomonoecious plants in all populations. The frequency of sexual morphs varied significantly among the studied populations but not among species. Female plants generally produced smaller numbers of flowers than hermaphroditic or gynomonoecious plants, and the percentages of female flowers per population were low. All these findings suggest that the gynodioecious–gynomonoecious sexual system in section *Psammophilae* is closer to hermaphroditism or gynomonoecy than gynodioecy.

## Abstract (precis)

Species or populations may be categorized by sexual system. Here, we examine the frequency of sexual systems in *Silene*. We found that hermaphroditism is the most common sexual system, followed by dioecy, gynodioecy and gynodioecy-gynomonoecy (females, hermaphrodites and gynomonoecious plants) with similar frequency. These sexual systems are equally represented in the two phylogenetically supported subgenera *Silene* and *Behenantha*. We specifically studied the sexual systems of section *Psammophilae* (four species and 26 populations), and found that most populations are gynodioecious-gynomonoecious. Hermaphrodites are the most common sexual morph, and females tend to produce fewer flowers than other morphs.

## Introduction

The study of the diversity and evolution of sexual systems in plants has been the focus of many scientists since early days. Species or populations may be categorized by sexual system, depending on the spatial distribution of male and female reproductive structures within and among plants (Bawa and Beach 1981). Although many different sexual systems may exist (Sakai and Weller 1999), most of the angiosperm species belong to one of the five main types: hermaphroditism (72 %), gynodioecy (7 %, female and hermaphroditic individuals), monoecy (5 %, individuals with female and male flowers), dioecy (5–6 %, female and male individuals) and gynomonoecy (3 %, individuals with female and hermaphroditic flowers) (Richards 1997; Renner 2014). Although only 5–6 % of total angiosperms are dioecious, dioecious species are present in 43 % of families, and from 871 to 5000 independent origins of dioecy have been proposed (Renner 2014). Therefore, the evolutionary pathways to dioecy have been the focus of interesting debate, specially the transition from hermaphroditism to dioecy, with gynodioecy or monoecy as intermediate steps (Charlesworth 1999). The association of gynodioecy or monoecy with dioecy at the family or genera level suggests that both are possible pathways to dioecy (Renner and Ricklefs 1995; Dufay *et al.* 2014; Renner 2014). Gynomonoecy occurs frequently in families such as Compositae or Chenopodiaceae (Yampolsky and Yampolsky 1922; Torices *et al.* 2011), and has been considered the main route to monoecy from hermaphroditism and vice versa (Torices *et al.* 2011).

The genus *Silene* (Caryophyllaceae) has been widely used to study the evolution of sexual systems and gender variation (Meagher 2007; Bernasconi *et al.* 2009; Charlesworth 2013; Weingartner and Delph 2014), and is one of the groups used for the phylogenetic approach (Desfeux *et al.* 1996; Rautenberg *et al.* 2010; Marais *et al.* 2011). Nonetheless, the complete phylogenetic relationship within this genus is not yet resolved (Rautenberg *et al.* 2010; Petri *et al.* 2013). What seems clear is the subdivision of *Silene* (*sensu* Oxelman and Lidén 1995) into two clades: subgenera *Silene* and *Behenantha* (Popp and Oxelman 2004, 2007; Rautenberg *et al.* 2010). The first of these phylogenetic studies found that dioecy appeared independently at least twice (in subsection *Otites* and section *Melandrium*; according to Oxelman *et al.* 2013), and that gynodioecy was the most probable ancestral condition for the genus (Desfeux *et al.* 1996). More recently, Marais *et al.* (2011) found that either gynodioecy or hermaphroditism could be the ancestral condition of *Silene*. In *Otites* and *Melandrium*, different types of sex-determining systems with a different date of origin are implicated (Käfer *et al.* 2013; Slancarova *et al.* 2013). In addition to dioecy, hermaphroditism and gynodioecy are common in *Silene* (Desfeux *et al.* 1996; Jürgens *et al.* 2002). However, monoecy is not present, suggesting the evolution of dioecy through the gynodioecy pathway.

Gynomonoecy, andromonoecy (individuals with male and hermaphroditic flowers) and trioecy (populations with hermaphroditic, male and female individuals) have also been reported for *Silene*, but are very rare (Desfeux *et al.* 1996; Jürgens *et al.* 2002; present study). However, in a non-negligible number of gynodioecious species, the existence of gynomonoecious individuals (i.e. plants with female and hermaphroditic flowers) in the populations is reported (e.g. Shykoff 1988; Talavera *et al.* 1996; Lafuma and Maurice 2006; Dufay *et al.* 2010). Species or populations containing hermaphroditic, female and gynomonoecious individuals must be considered as gynodioecious–gynomonoecious (Gd–Gm hereafter) (Desfeux *et al.* 1996). The frequency of gynomonoecious plants may be highly variable among populations and species; in some cases, this sexual morph is rare and in others it may be the most frequent (Charlesworth and Laporte 1998; Maurice 1999; Dufay *et al.* 2010; Casimiro-Soriguer *et al.* 2013). The genetic mechanism for sex determination of gynodioecy may be based on the interaction of cytoplasmic male sterility genes with nuclear restorers of male fertility (Bailey and Delph 2007), as found in *S. vulgaris* (Charlesworth and Laporte 1998). In some cases, the incomplete restoration of the cytoplasmic male sterility factors or heteroplasmy (the occurrence of different cytotypes within an individual) can cause partially male-sterile plants that are able to produce females and hermaphroditic flowers (i.e. gynomonoecious plants) (Koelewijn and Van Damme 1996; McCauley *et al.* 2005). Thus, although the genetic basis for gynomonoecious and female individuals in Gd–Gm species has been hypothesized in *Silene* species (Glaettli and Goudet 2006; Garraud *et al.* 2011), their incidence remains unclear.

*Silene littorea* is one of the most studied species with a Gd–Gm sexual system (Guitián and Medrano 2000; Vilas and García 2006; Vilas *et al.* 2006; Casimiro-Soriguer *et al.* 2013). In several populations from two contrasting sites in their distribution area, the frequency of hermaphrodites or gynomonoecious plants varied highly among populations, but female plants were always rare (Guitián and Medrano 2000; Casimiro-Soriguer *et al.* 2013). Analysis of functional gender showed that nearly all plants in the population transmit their genes via both pollen and ovules; thus, the Gd–Gm sexual system of *S. littorea* seems to be closer to hermaphroditism or gynomonoecy than gynodioecy (Casimiro-Soriguer *et al.* 2013). Interestingly, *S. stockenii* also shows a Gd–Gm sexual system with a very low frequency of female plants (Talavera *et al.* 1996). Both species belong to the section *Psammophilae*, composed of three other annual species (*S. adscendens*, *S. cambessedesii* and *S. psammitis*). Therefore, the question which arises from these findings is whether the Gd–Gm sexual system is widespread in the whole *Psammophilae* section. In addition, the reproductive output of the different morphs may vary in the Gd–Gm sexual system, which may be important to the stable maintenance of these morphs in the populations (Dufay *et al.* 2010). For instance, Shykoff *et al*. (2003) found that overall females produce more but smaller flowers, set more fruits and produce more and heavier seeds than hermaphrodites.

In this study, two different approaches were used to evaluate the occurrence of sexual systems, particularly gynodioecy–gynomonoecy, in *Silene*. For the general approach, we searched the literature extensively to locate any direct or indirect description of the sexual system of the species of *Silene*. This search allows us to know the frequency of sexual systems at the genus and infrageneric level as well as their variability within species. Accurate estimates of the frequency of the Gd–Gm sexual system may shed light on their possible evolution and stability in *Silene*, and also in other groups of angiosperms. For the specific approach, we have studied the sexual systems of a total 26 populations of the species of the section *Psammophilae*. Specifically, we seek to answer the following questions. (i) Is the Gd–Gm sexual system widespread throughout the distribution area of *S. littorea* and the other species of section *Psammophilae*? (ii) What is the frequency of the different sexual morphs and types of flowers in the populations? (iii) Are there differences in the number of flowers produced by each morph?

## Methods

### Study system

*Silene littorea*, *S. cambessedesii*, *S. psammitis* and *S. stockenii* are endemic to the Iberian Peninsula and Balearic Islands (Talavera 1979). Talavera (1979) included these taxa together with *S. almolae*, *S. germana* and *S. pendula* within the section *Erectorefractae*. We follow Greuter (1995) who proposed the section *Psammophilae*, previously considered a subsection of *Erectorefractae* (Talavera 1979). Oxelman *et al.* (2013) consider the species status of *S. adscendens* (previously considered a subspecies of *S. littorea*). All the species are spring-flowering annuals and grow in different types of soil: sandy substrates from the coast (*S. cambessedesii*, *S. littorea*), dolomites or slates (*S. psammitis*), calcareous sandstones (*S. stockenii*) or schists (*S. adscendens*) (Talavera 1979).

### Analysis of the sexual system of section *Psammophilae*

**Figure 1.**
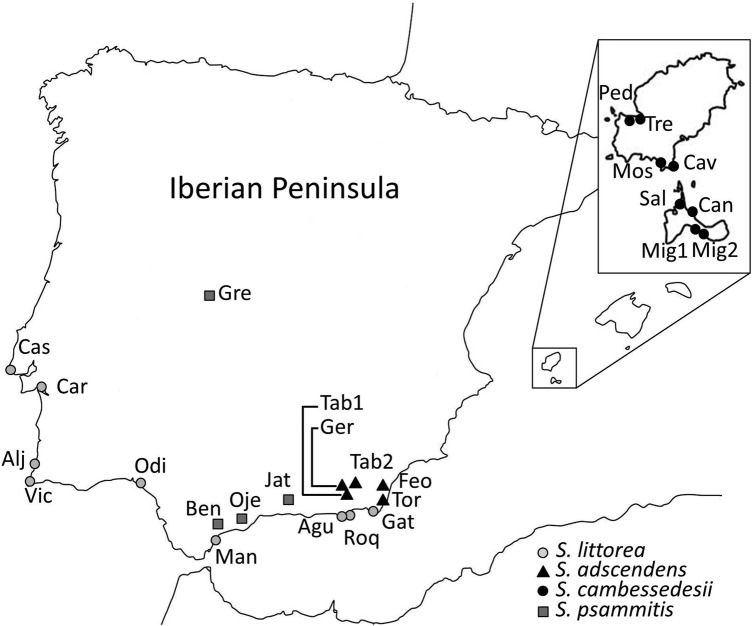
Populations sampled from the different species of section *Psammophilae*: nine populations of *S. littorea* (grey dots), five populations of *S. adscendens* (black triangles), eight populations of *S. cambessedesii* (black dots) and four populations of *S. psammitis* (grey squares).

During the peak of the flowering period from 2010 to 2012, we visited 5 populations of *S. adscendens*, 8 of *S. cambessedesii*, 11 of *S. littorea* and 4 of *S. psammitis* (Fig. 1) **[see Supporting Information]**. We did not include *S. stockenii* because: (i) it is a critically endangered species with only a few populations (Bañares *et al*. 2004), (ii) Talavera *et al.* (1996) have already studied the sexual system of *S. stockenii* in the most important population and (iii) we visited some of the remnant populations, detecting high florivory levels and a small number of individuals. We performed single-day linear transects of 100 plants, with the exception of some very small populations **[see Supporting Information]**. We chose plants separated by at least 1 m to avoid microhabitat or clustering effects in sex expression (Klaas and Olson 2006). For each plant, we counted all the flowers in anthesis, and noted their sex (female or hermaphroditic). Withered flowers were also analysed when sex differentiation was possible. Plants bearing only female or hermaphroditic flowers were considered female or hermaphroditic individuals, respectively, whereas individuals with female and hermaphroditic flowers were considered gynomonoecious. In a previous study in *S. littorea*, Casimiro-Soriguer *et al.* (2013) found that the probability of recording female flowers in gynomonoecious plants was higher when the whole flowering period of a plant was studied than when estimates were based on a single census. Thus, our sampling methodology would underestimate the frequency of gynomonoecious plants in the population. In addition, plants with a large number of flowers would have a higher probability to be classified as gynomonoecious.

### Literature search on Silene sexual systems

We performed a literature search in the SCOPUS and JSTOR databases including the terms: *Silene*, breeding system, sexual system, hermaphroditic, hermaphrodite, hermaphroditism, dioecious, dioecy, gynodioecious, gynodioecy, gynomonoecious, gynomonoecy, androdioecious, androdioecy, andromonoecious, andromonoecy, monoecious and monoecy. We also revised the description of the *Silene* species in main floras and revision studies or books previous to 1938 that contain information about plant sexuality, see Table 1 and **[Supporting Information]**. In addition, for those species with numerous sexual system descriptions or present in the Euro + Med database, specific individual searches were performed. We annotated the information about the sexual systems of the species in one of the following categories: hermaphrodite (H), dioecious (D), gynodioecious (Gd), gynomonoecious (Gm), androdioecious (Ad), andromonoecious (Am) or trioecious (T). In some cases, mixed sexual systems were found within a single population, for instance Am–Ad (male, hermaphroditic and andromonoecious plants), H–Gm (hermaphrodites and gynomonoecious plants) and gynodioecious–gynomonoecious (Gd–Gm, female, hermaphroditic and gynodioecious plants). When various studies described a species with different sexual systems, all of them were annotated with the respective reference; however, the principal or most frequent sexual system was used for calculating the frequency at the genus or subgenus level (see Jürgens *et al.* 2002 for similar criteria). The H–Gm category was assigned as H because in most cases gynomonoecious individuals are extremely rare and bear only a few female flowers (e.g. A. Jürgens, pers. comm.; Giménez-Benavides *et al.* 2007). We will follow the classification criteria of Oxelman *et al.* (2013) for *Silene* and the infrageneric level. The subspecies level was not considered.

**Table 1.** Sexual systems in *Silene*. Sexual system description recognizes all the sexual systems described for the species in the literature. The sexual system assigned here is the principal or most frequent sexual system for the species according to our review. Species classification follows Oxelman *et al.* (2013). H, hermaphrodite; D, dioecious; Gd, gynodioecious; Gm, gynomonoecious; Ad, androdioecious; Am, andromonoecious and T, trioecious. Mixed sexual systems are denoted by a dash.

Subgenus, section, species	Sexual system	
	Described in literature	Assigned
**Subgenus *Behenantha*** (Otth) Endl.		
Section *Behenantha* Otth		
*S. pendula* L.	Gd^1,2,51^	Gd
*S. uniflora* Roth	Gd^3,4,5^	Gd
*S. vulgaris* (Moench) Garcke	Am–Ad^6^; Gd^2^; Gd–Gm^6,7,8,9,10^	Gd–Gm
Section *Conoimorpha* Otth		
*S. conica* L.	H^2,7^; H–Gm^2^	H
*S. conoidea* L.	H^1,2^	H
*S. subconica* Friv.	Gd^2^	Gd
Section *Dichotomae* (Rohrb.) Chowdhuri		
*S. dichotoma* Ehrh.	Gd^2,6,7^	Gd
Section *Elisanthe* (Fenzl) Fenzl		
*S. noctiflora* L.	H^11^;H–Gm^2,7^;Gm^12,13^;Gd–Gm^1^	Gm
Section *Erectorefractae* Chowdhuri		
*S. germana* Gay	H^51^	H
Section *Melandrium* (Röhl.) Rabeler		
*S. astrachanicum* (Pacz.) Takht.	D^14^	D
*S. diclinis* (Lag.) M.Laínz	D^1,8,14,15,16,17^	D
*S. dioica* (L.) Clairv.	Am^6^; D^2,6,7,8,14,15^	D
* S. integripetala* Bory and Chaub.	H–Gm^30^	H
Section *Viscosae* (Boiss.) C.L.Tang		
* S. viscosa* (L.) Pers.	H^2,7^	H
Others		
* S. acutifolia* Link ex Rohrb.	H^8,31^	H
* S. elisabethae* Jan	H^6,7^	H
**Subgenus *Silene***		
Section *Auriculatae* (Boiss.) Schischkin		
* S. disticha* Willd.	H^2,8^	H
* S. echinata* Otth	H^8^; H–Gm^2^	H
* S. linicola* C.C.Gmel.	H^2,6,8^	H
* S. schafta* J.G.Gmel. ex Hohen.	Gd^2^	Gd
* S. spergulifolia* (Willd.) M.Bieb.	H^2^	H
* S. vallesia* L.	Gd^2,7^	Gd
Section *Silene*		
* S. apetala* Willd.	H^1,2^	H
* S. ciliata* Pourr.	H–Gm^32^	H
* S. colorata* Poir.	H^8,33^; H–Gm^2^	H
* S. gallica* L.	H^8^; H–Gm^2^	H
* S. gracilis* DC.	H^51^	H
* S. micropetala* Lag.	H^8^; H–Gm^2^	H
* S. nicaeensis* All.	H^2,8,51^	H
* S. nocturna* L.	H^1,2,8^	H
* S. pseudoatocion* Desf.	Gd–Gm^2^	Gd–Gm
* S. ramosissima* Desf.	H^8,51^	H
* S. scabriflora* Brot.	H^51^	H
* S. roemeri* Friv.	Gd–Am^15^; H–Gd^2^	Gd
* S. saxifraga* L.	Am–Gm–T^6^; Gm^1^; Gd–Gm^2,7^	Gd–Gm
* S. sendtneri* Boiss.	D^15^	D
* S. senneni* Pau	H^45^	H
* S. thessalonica* Boiss. and Heldr.	H–Gm^2^	H
* S. viridiflora* L.	H^7^; Gd–Gm^2^	Gd–Gm
* S. waldsteinii* Griseb.	H–Gm^2^	H
* S. wolgensis* (Hornem.) Otth	D^36^	D
Section *Spergulifoliae* (Boiss.) Schischkin		
* S. brahuica* Boiss.	Gd^21^	Gd
Others		
* S. bupleuroides* L.	H^2,7^	H
*S. heuffelii* Soó	D^14^	D
*S. latifolia* Poir.	Am^6^; D^2,6,7,8,14,15^	D
*S. marizii* Samp.	D^8^	D
Section *Physolychnis* (Bentham) Bocquet		
*S. caroliniana* Walter	H^18^	H
*S. douglasii* Hook.	H^11,19^	H
*S. gangotriana* Pusalkar, D.K.Singh and Lakshmin	H^20^	H
*S. laxantha* Majumdar	Gd^20,21^	Gd
*S. regia* Sims	H^22,23^	H
*S. rotundifolia* Nutt.	H^23^	H
*S. scouleri* Hook.	H^11^	H
*S. stellata* (L.) W.T. Aiton	H^18,24^	H
*S. tibetica* Lidén and Oxelman	H–Am^25^	Am
*S. virginica* L.	H^18,26^	H
*S. zawadzkii* Herbich	H^2^	H
Section *Psammophilae* (Talavera) Greuter		
*S. adscendens* Lag.	Gd–Gm^50^	Gd–Gm
*S. cambessedesii* Boiss. and Reut.	Gd–Gm^50^	Gd–Gm
*S. littorea* Brot.	Gd–Gm^2,27,28,50^	Gd–Gm
*S. psammitis* Link ex Spreng	Gd–Gm^50^	Gd–Gm
*S. stockenii* Chater	Gd–Gm^29,51^	Gd–Gm
Section *Sedoideae* Oxelman and Greuter		
*S. secundiflora* Otth	H^2,8^	H
*S. sericea* All.	Gd^2^	Gd
*S. succulenta* Forssk.	H^2^	H
Section *Siphonomorpha* Otth		
*S. acaulis* (L.) Jacq.	D^2,6,7,14^; T^6,7,15,34^; Gd–Gm^35^	D
*S. andryalifolia* Pomel	Gd^2^	Gd
*S. borysthenica* (Gruner) Walters	D^15,36^	D
*S. colpophylla* Wrigley	D^37^	D
*S. cyri* Schischkin	D^14^	D
*S. fernandezii* Jeanm.	H–Gm^8^	H
*S. flavescens* Waldst. and Kit.	H–Gm^2^	H
*S. fruticosa* L.	H^2^	H
*S. gazulensis* Galán, Cortés, Orell. and Morales Alonso	H^38^	H
*S. gigantea* L.	H–Gm^39^	H
*S. hayekiana* Hand.-Mazz. and Janch.	Gd^2^	Gd
*S. hellmannii* Claus	D^14,15^	D
*S. hifacensis* Rouy	Gd^23^; Gd–Gm^40^	Gd–Gm
*S. italica* (L.) Pers.	H^7^; Gd^2^; Gd–Gm^1,41,42^	Gd–Gm
*S. multicaulis* Guss.	H^2^	H
*S. multiflora* (Ehrh) Pers.	H^7^	H
*S. nocteolens* Webb and Berthel.	H^38^	H
*S. nutans* L.	Am–Ad^6^; Gd–Gm^1,2,6,7,43^	Gd–Gm
*S. otites* (L.) Wibel	Ad^6^; D^1,2,6,7,15,36^	D
*S. paradoxa* L.	H^2,23^	H
*S. parnassica* Boiss. and Spruner	H^2^	H
*S. patula* Desf.	H^44^	H
*S. capitellata* Boiss.	H^46^	H
*S. chlorantha* (Willd.) Ehrh	H^2,7,47^	H
*S. cretica* L.	H^2,7,8^	H
*S. friwaldskyana* Hampe.	H^2^	H
*S. hawaiiensis* Sherff	H^23,48^	H
*S. inaperta* L.	H^1,2^	H
*S. isaurica* Contandr. and Quézel	Gd^46^	Gd
*S. kemoniana* C. Brullo, Brullo, Giusso, Ilardi and Sciandr.	H^49^	H
*S. muscipula* L.	H^2^	H
*S. portensis* L.	H^2,51^	H
*S. struthioloides* A.Gray	H^23,48^	H

^1^Desfeux *et al*. (1996), ^2^Jürgens *et al*. (2002), ^3^Baker and Dalby (1980), ^4^Pettersson (1997), ^5^Warren and James (2008), ^6^Knuth (1908), ^7^Meusel and Mühlberg (1979), ^8^Talavera (1990), ^9^Glaettli and Goudet (2006), ^10^Miyake and Olson (2009), ^11^Touzet and Delph (2009), ^12^Folke and Delph (1997), ^13^Davis and Delph (2005), ^14^Schischkin (1970), ^15^Chater and Walters (1964), ^16^Prentice (1976), ^17^Montesinos *et al*. (2006), ^18^Reynolds *et al*. (2009), ^19^Kephart *et al*. (1999), ^20^Pusalkar *et al*. (2004), ^21^Tropicos.org (2014), ^22^Dolan (1994), ^23^Moyle (2006), ^24^Castillo *et al*. (2013), ^25^Oxelman *et al*. (2001), ^26^Dudash and Fenster (2001), ^27^Guitián and Medrano (2000), ^28^Casimiro-Soriguer *et al*. (2013), ^29^Talavera *et al*. (1996), ^30^Oxelman (1995), ^31^Buide and Guitián (2002), ^32^Giménez-Benavides *et al*. (2007), ^33^Terrab *et al*. (2007), ^34^Alatalo and Molau (2001), ^35^Shykoff (1992), ^36^Lihua *et al*. (2001), ^37^Mrackova *et al*. (2008), ^38^Bañares *et al*. (2004), ^39^Ghazanfar (1989), ^40^Prentice *et al*. (2003), ^41^Maurice (1999), ^42^Lafuma and Maurice (2006), ^43^Dufay *et al*. (2010), ^44^Naciri *et al*. (2010), ^45^Martinell *et al*. (2010), ^46^Yildiz and Çirpici (2013), ^47^Lauterbach *et al*. (2011), ^48^Westerbergh and Saura (1994), ^49^Brullo *et al*. (2012), ^50^Casimiro-Soriguer *et al*. present study and ^51^E. Narbona, M. L. Buide and I. Casimiro-Soriguer, pers. observations.

### Statistical analysis

To test for differences in the proportion of the different sexual morphs among species and populations, a generalized linear model (GLM) with a multinomial distribution and a probit link function was carried out. We considered the sexual morph of each individual (female, hermaphrodite or gynomonoecious) as the multinomial response variable; and species and population (nested within species) as fixed factors. Population was treated as a fixed factor rather than a random factor because we are interested in examining the differences in morph frequencies among our specific populations, and the same populations would be analysed in future studies (Bennington and Thayne 1994; Potvin 2001). Comparisons of the number of flowers between female plants and hermaphrodite or gynomonoecious plants were performed using GLMs with a log link function and a Poisson error distribution. The dependent variable was the number of flowers produced by each individual; and sexual morph, population (nested within species) and species were included as fixed factors. On the other hand, the frequency of each sexual system between the subgenus *Silene* and *Behenantha* was compared using χ^2^ tests for contingency tables (Quinn and Keough 2002). All the analysis were carried out in IBM^®^ SPSS^®^ Statistics v.22.

## Results

### Sexual system of the section *Psammophilae*

**Figure 2.**
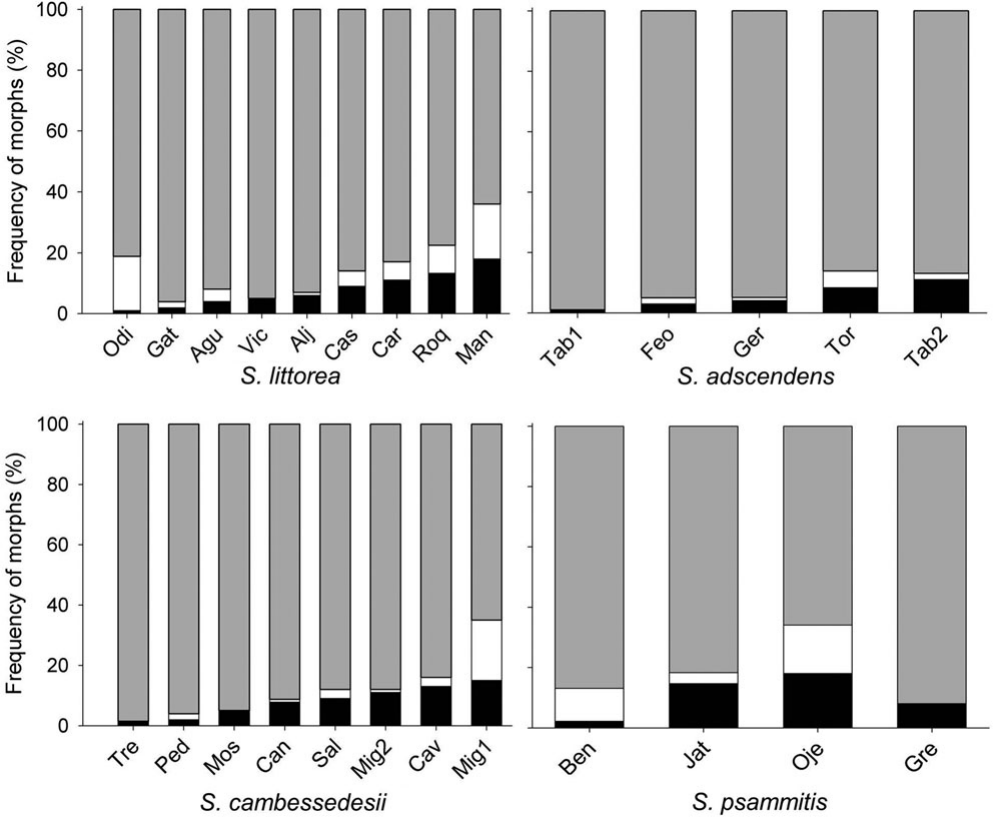
Frequency of hermaphroditic (grey), gynomonoecious (white) and female (black) individuals of species from section *Psammophilae* in each population. The number of individuals per sexual morph sampled in each population is shown elsewhere [**see Supporting Information**].

A total of 2478 individuals belonging to 26 populations were surveyed. In general, each studied taxa of section *Psammophilae* showed Gd and Gd–Gm populations, although Gd–Gm populations were the most frequent (Fig. 2). *Silene littorea*, *S. adscendens* and *S. psammitis* showed one Gd population each, whereas *S. cambessedesii* showed two Gd populations. The remaining populations were all Gd–Gm (Fig. 2).

Overall, the most frequent morph of section *Psammophilae* was the hermaphrodite, with an 86.8 % of individuals included in this category, followed by the female and the gynomonoecious morphs (7.9 and 5.3 %, respectively). The proportion of hermaphroditic plants within populations ranged from 64.0 % (in *S. littorea*) to 99 % (in *S. adscendens*), whereas the proportion of female plants varied from 1.0 to 18.0 % (in *S. littorea*), and that of gynomonoecious plants ranged from zero (at least one population in each species) to 20 % (in *S. psammitis*) (Fig. 2). The frequency of sexual morphs per population varied significantly among the studied populations (Wald χ^2^ = 62.62, df = 22, *P* < 0.0001) but not among species (Wald χ^2^ = 4.59, df = 3, *P* = 0.21).

On the whole, 92.1 % of the 7001 flowers analysed were hermaphrodites, and 7.9 % were females. At the species level, *S. psammitis* showed the highest proportion of female flowers (16.1 %) in the population and *S. adscendens* the lowest (4.8 %) **[see Supporting Information]**. The predominance of hermaphrodite flowers was also found at the population level; the mean percentage of female flowers per population ranged from 1.3 to 18.7 % in *S. littorea*, from 0.8 to 10.2 % in *S. adscendens*, from 1.1 to 14.1 % in *S. cambessedesii* and from 7.1 to 29.4 % in *S. psammitis* **[see Supporting Information]**. On the other hand, the percentage of female flowers in gynomonoecious individuals was 34.8 ± 2.0 % (mean ± 1 SE) in *S. littorea*, 34.5 ± 5.2 % in *S. adscendens*, 31.4 ± 3.0 % in *S. cambessedesii* and 43.4 ± 3.1 % in *S. psammitis* **[see Supporting Information]**.

The average number of flowers in female plants was generally smaller than in hermaphroditic or gynomonoecious plants **[see Supporting Information]**. The number of flowers per individual showed significant differences among sex morphs (Wald χ^2^ = 328.85, df = 2, *P* < 0.0001), populations (Wald χ^2^ = 2126.73, df = 22, *P* < 0.0001) and species (Wald χ^2^ = 571.88, df = 3, *P* < 0.0001).

### Diversity and frequency of sexual systems in *Silene*

**Figure 3.**
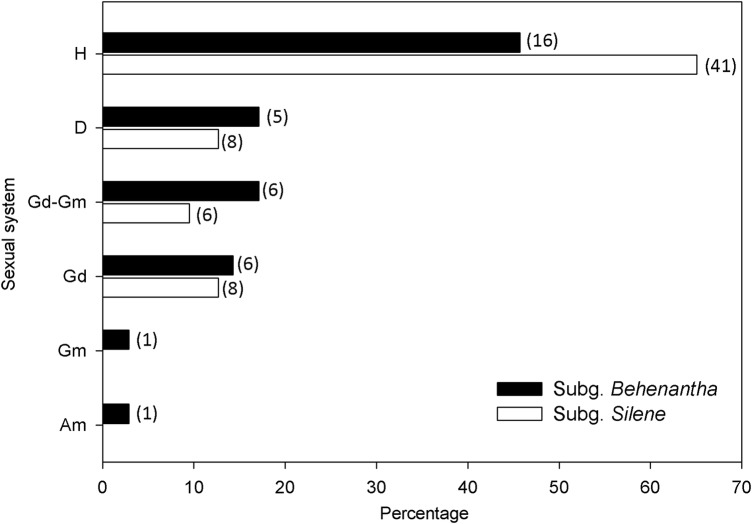
Proportion of sexual systems in subgenus *Behenantha* (black bars) and subgenus *Silene* (white bars). H, Hermaphroditism; D, dioecy; Gd–Gm, gynodioecy–gynomonoecy; Gd, gynodioecy; Gm, gynomonoecy; Am, andromonoecy.

We found that 98 *Silene* species have been specifically studied or described in terms of the sexual system (Table 1). We have collected the data from 46 different species in addition to those formerly found by Desfeux *et al*. (1996) and Jürgens *et al.* (2002). The number of species described in the subgenus *Silene* (63 species) is nearly double that in subgenus *Behenantha* (35 species) (Table 1). The most frequent sexual system at the genus level is hermaphroditism (58.2 %), followed by dioecy (14.3 %), gynodioecy (13.3 %) and gynodioecy–gynomonoecy (12.2 %). Interestingly, all four sexual systems are present in both subgenera, with a statistically similar frequency (*P* > 0.43 for all the sexual systems, except for hermaphroditism, that showed marginally significant differences *P* = 0.09) (Fig. 3). In addition, one Gm and one Am species were found, and both belong to the subgenus *Behenantha*. The fact that 13 of our assigned H species (21.1 %) are described as H–Gm in the literature is worthy of mention.

There are some sections whose species present mainly the same sexual system. For instance, section *Melandrium* are all dioecious, section *Psammophilae* are all Gd–Gm and section *Physolychnis* are all hermaphroditic except one species (*S. laxantha*, Table 1). Other sections seem more variable. Thus, section *Silene* includes H, Gd and Gd–Gm species, and section *Siphonomorpha* includes all the sexual systems present in the subgenus; however, it can be said that both sections have the highest number of species with known sexual systems.

In the literature, we have found 17 species (17.3 %) whose description of the sexual system varies. For instance, *S. noctiflora* has been described as H, H–Gm, Gm or Gd–Gm, and *S. acaulis* as D, T or Gd–Gm. By contrast, in other species, the sexual system description is consistently confirmed by several studies (e.g. the dioecious *S. diclinis* or the hermaphroditic *S. chloranta*).

## Discussion

Studies of sexual systems over an entire section of *Silene* are mostly focussed on those groups containing dioecious species (Marais *et al.* 2011; Slancarova *et al.* 2013). To the best of our knowledge, this is the first study that analyses the sexual system of a whole section in *Silene* composed of non-dioecious species, including multiple populations across their distribution area. All species of section *Psammophilae* should be considered Gd–Gm, despite their traditional description as hermaphrodites (Talavera 1990; Greuter 1995). The Gd–Gm sexual system has been described in other species of the Caryophyllaceae family (e.g. *Dianthus sylvestris*, *Gypsophila repens*, *Stellaria longipes*; Philipp 1980; Collin and Shykoff 2003; López-Villavicencio *et al.* 2005). However, their presence in other families seems scarce; only a few cases are known in the Plantaginaceae and Lamiaceae (Kheyr-Pour 1980; Koelewijn and Van Damme 1996; Widén and Widén 1999).

We have found that hermaphroditic plants are the most frequent morph in all populations and species, including other species in the section *Psammophilae*, *S. stockenii* (Talavera *et al.* 1996). Similar results have been found in other Gd–Gm species of *Silene*, such as *S. italica* and *S. nutans* (Maurice 1999; Dufay *et al.* 2010). We also have demonstrated that the proportion of gynomonoecious plants varies among populations. Other previously studied populations of *S. littorea* showed hermaphroditic individuals in a similar or smaller frequency than the gynomonoecious (Guitián and Medrano 2000; Casimiro-Soriguer *et al.* 2013). This fact may be explained by this high inter-population variability in the frequency of hermaphroditic plants, but variations due to the different sampling methodology cannot be excluded. At least in *S. littorea* it is possible that plants classified as hermaphrodites in a single-census day could produce a female flower throughout the flowering period (Casimiro-Soriguer *et al.* 2013).

Most *Silene* species reviewed here were hermaphroditic (ca. 60 %), but dioecious, gynodioecious and Gd–Gm species were also relatively frequent. Interestingly, this genus was considered predominantly gynodioecious by some authors (Matsunaga and Kawano 2001; Lengerova *et al.* 2003; Slancarova *et al.* 2013). Knuth (1908) reported the presence of androdioecy or andromonoecy in several species, but this has never been confirmed in further studies. More recently, Oxelman *et al.* (2001) described the presence of apparently functionally male flowers in the lateral positions of the dichasium in *S. tibetica*, but again no further studies have confirmed this finding. On the other hand, hermaphroditism, dioecy, gynodioecy and Gd–Gm are present in both subgenus *Behenantha* and *Silene* at similar frequencies. The presence of each sexual system in both phylogenetically supported subgenera suggests a repeated independent evolution of sexual systems in these *Silene* clades, as found in other groups (Renner and Won 2001; Soza *et al*. 2012). In fact, repeated evolution of dioecy is phylogenetically confirmed in *Silene* (Marais *et al*. 2011; Slancarova *et al.* (2013).

Our survey of sexual systems in *Silene* showed that although most species seem to be consistent in their sexual system, 17 % of the reported species were described with more than one sexual system. This variation may be caused by different authors assigning sexual systems [e.g. *S. dioica* and *S. latifolia* are dioecious, but have been considered andromonoecious by Knuth (1908)] or by authors' simplification due to the low frequency of some sexual morphs in populations. However, in most cases, these differences could correspond to variations within or among populations (e.g. *S. acaulis*, *S. noctiflora*, *S. saxifraga* and *S. vulgaris*; see references in Table 1). This variation may be related to the genetic basis of sex determination and/or ecological factors acting on sexual expression (Delph 2003; McCauley and Bailey 2009). For example, the sexually plastic *S. acaulis* shows dioecy, trioecy, gynomonoecy or gynodioecy across its distribution area (Maurice *et al.* 1999; Alatalo and Molau 2001; Delph and Carroll 2001) and a cytoplasmic determination of sex with nuclear male fertility restorer genes is suggested (Delph *et al.* 1999; Klaas and Olson 2006). In addition, the role of environmental factors in sex expression has also been demonstrated in different species with higher frequency of female plants in harsher or dryer environments (Delph 2003). For instance, a higher female frequency in low-quality sites was found in *S. acaulis* (Delph and Carroll 2001).

A question arising from the relative high frequency of gynodioecy–gynomonoecy in *Silene*, and particularly in section *Psammophilae*, is whether this sexual system is an evolutionarily stable strategy. Theoretical models suggest that gynodioecy can evolve into dioecy, but also can be stable (Charlesworth and Charlesworth 1978; Dufay *et al.* 2014). Less is known about the maintenance of gynomonoecy (De Jong *et al.* 2008; Mamut *et al.* 2014), and especially Gd–Gm (McCauley and Bailey 2009; Garraud *et al.* 2011). In an evolutionarily stable Gd–Gm sexual system, female and gynomonoecious individuals must compensate for their loss of male function at the individual and flower level, respectively (Lloyd 1984). In gynodioecy, the advantage of female plants over hermaphrodites can be through inbreeding avoidance, resource reallocation or sex difference interactions with herbivores (Ashman 2002; Dufay and Billard 2012). The degree of female advantage should have an impact on the frequency of females (Dufay *et al.* 2007). In that case, those species with low female advantage will have low or variable frequency of female plants (Dufay *et al.* 2014). We found a low frequency of female plants per population in all species of section *Psammophilae*, as well as in the Gd–Gm species *S. italica* and *S. nutans* (Maurice 1999; Dufay *et al.* 2010). In these species, female advantage over hermaphrodites due to reallocation of resources seems to be low (Lafuma and Maurice 2006; Dufay *et al.* 2010). For instance, in *S. nutans* there were no differences in seed mass, germination rate or offspring quality between females and hermaphrodites (Dufay *et al*. 2010). In *S. stockenii*, females produced similar fruit set and number of seeds to hermaphroditic or gynomonoecious plants (Talavera *et al.* 1996). Similarly, in *S. littorea* female plants set similar fruits than gynomonoecious or hermaphrodites plants (Guitián and Medrano 2000). We have found that the number of flowers in female plants was smaller than that in the other morphs in some of the populations analysed. Thus reproductive output of female plants in the section *Psammophilae* seems to be lower than those of gynomonoecious or hermaphrodites, but further studies are needed to assess the possible female advantage in these species. On the other hand, the avoidance of inbreeding depression by female plants of *S. littorea* could help to maintain this morph in the population, although in a low frequency (Vilas and García 2006).

With regard to reproductive compensation of gynomonoecious plants over hermaphrodites, three main hypotheses have been proposed: (i) two types of flowers may allow the reallocation of resources to male and female functions (Lloyd 1979), (ii) female flowers can partially avoid inbreeding depression by favouring outcrossing (Marshall and Abbott 1984; Mamut *et al.* 2014) and (iii) flowers can escape florivory since hermaphrodites are usually more often attacked (Ashman 2002; Bertin *et al.* 2010). The outcrossing–benefit hypothesis of gynomonoecy has been demonstrated in *Eremurus anisopterus* (Mamut *et al*. 2014) and in *S. noctiflora* (Davis and Delph 2005). In the former, perfect flowers promote seed quantity by increasing pollinator attraction, whereas in the latter perfect flowers provide reproductive assurance by autonomous selfing when pollinators are scarce. As *Silene* species are self-compatible, autogamous selfing is possible where there is an overlap between sexual phases in the protandrous hermaphroditic flowers (Davis and Delph 2005; M. L. Buide, unpubl. data). In three populations of *S. littorea*, around 20 % of seed set was due to autonomous selfing (Hidalgo-Triana 2010), with similar findings for *S. stockenii* (23 %; Talavera *et al.* 1996). Thus, in these species of section *Psammophilae*, perfect flowers in gynomonoecious plants could allow some levels of reproductive assurance, whereas female flowers could partially avoid inbreeding depression. On the other hand, environmental factors could also affect the production of female flowers in gynomonoecious plants, and consequently affect sex expression in species of section *Psammophilae*. In the gynomonoecious *S. noctiflora*, an increase of 6 °C in a greenhouse, increased the production of female flowers in gynomonoecious plants (Folke and Delph 1997).

## Conclusions

To sum up, we have confirmed the high diversity of sexual systems in *Silene*, but we have also demonstrated that the most important sexual systems are similarly represented in both subgenera *Silene* and *Behenantha*. The Gd–Gm sexual system is found in a similar number of species as dioecy and gynodioecy. In addition, we have documented that most populations of species from section *Psammophilae* showed a Gd–Gm sexual system, but variations in sexual expression also exist. The low number of females and gynomonoecious plants, and the low percentage of female flowers at the population level, suggest that the Gd–Gm sexual system in section *Psammophilae* is closer to hermaphroditism or gynomonoecy than gynodioecy. Thus, our study generates an important question: Has the Gd–Gm sexual system any advantage over hermaphroditism and gynodioecy, or is it just a consequence of the genetic mechanism of gynodioecious sex determination? The main non-exclusive hypotheses proposed for the determination of the gynomonoecious morph are the effect of environmental factors, and the partial restoration of male fertility (Dufay *et al*. 2010 and references therein). However, to the best of our knowledge, explicit evolutionary models do not exist including the gynomonoecious plants and their role on evolutionary transitions (Garraud *et al.* 2011). Gd–Gm species of *Silene*, and especially those of the section *Psammophilae*, could be a good model system to study the maintenance of gynomonoecious individuals in Gd–Gm populations.

## Sources of Funding

This work was supported with FEDER funds and grants by the Spanish Ministerio de Ciencia e Innovación through a Formación de Personal Investigador grant to I.C.-S. [BES-2010-031073] and the research projects CGL2009-08257 and CGL2012-37646.

## Contributions by the Authors

E.N., M.L.B. and I.C.-S. conceived the idea and collected the field data. I.C.-S. performed the literature review. E.N. and I.C.-S. ran the statistics. I.C.-S. led the writing with assistance of the others.

## Conflict of Interest Statement

None declared.

## Supporting Information

The following additional information is available in the online version of this article –

**File S1.** Geographic coordinates and number of individuals analysed (N) in the populations of species from section *Psammophilae*.

**File S2.** Revised literature that was not cited in the manuscript because no information about the sexual system of species was found.

**File S3.** Percentage of female flowers (FF) in populations, average number of flowers per sexual morph and percentage of female flowers in gynomonoecious plants. Mean ± s.e. per species is highlighted in bold.

Additional Information
