# Supplementary material for: Diversity of sexual systems within different lineages of the genus Silene
Source: AoB Plants. 2015 May 15;7:plv037. doi: 10.1093/aobpla/plv037 (PMC4433491; doi:10.1093/aobpla/plv037)
Supplement: Additional Information [file supp_7_plv037_index.html]

Diversity of sexual systems within different lineages of the genus Silene — Diversity of sexual systems within different lineages of the genus Silene — Additional Information 

# Diversity of sexual systems within different lineages of the genus *Silene*

## Additional Information

Additional Information

**Files in this Data Supplement:**

- Supplementary File 1 - doc file
- Supplementary File 2 - docx file
- Supplementary File 3 - docx file
