# Supplementary material for: Diversity of sexual systems within different lineages of the genus Silene
Source: AoB Plants. 2015 May 15;7:plv037. doi: 10.1093/aobpla/plv037 (PMC4433491; doi:10.1093/aobpla/plv037)
Supplement: Additional Information [file supp_plv037_plv037supp_file1.doc]

**File 1**. Geographic coordinates and number of individuals analysed (N) in populations of species from section *Psammophilae*.

| **Taxon** | **Country** | **Population** | **Code** | **Latitude**  **(°N)** | **Longitude**  **(°E)** | **N** |
| --- | --- | --- | --- | --- | --- | --- |
| *S. littorea* | Spain | Cabo de Gata | Gat | 36.765550 | -2.230917 | 102 |
|  |  | Roquetas de Mar | Roq | 36.713056 | -2.635889 | 98 |
|  |  | San Agustin | Agu | 36.691028 | -2.701528 | 100 |
|  |  | Manilva | Man | 36.332278 | -5.239083 | 100 |
|  |  | Odiel | Odi | 37.164706 | -6.919111 | 101 |
|  | Portugal | Cabo San Vicente | Vic | 37.026394 | -8.991392 | 100 |
|  |  | Aljezur | Alj | 37.338767 | -8.851828 | 100 |
|  |  | Cascais | Cas | 38.702153 | -9.473942 | 100 |
|  |  | Carrasqueira | Car | 38.400822 | -8.710925 | 100 |
| *S. adscendens* | Spain | Gergal | Ger | 37.083361 | -2.507861 | 99 |
|  |  | Cala Los Toros | Tor | 36.822639 | -2.043222 | 72 |
|  |  | Los Feos | Feo | 37.013444 | -2.029278 | 100 |
|  |  | Tabernas1 | Tab1 | 37.007508 | -2.456094 | 100 |
|  |  | Tabernas2 | Tab2 | 37.115250 | -2.404389 | 100 |
| *S. cambessedesii* | Spain | Platja des Cavallet | Cav | 38.848139 | 1.401056 | 100 |
|  |  | Can Mosson | Mos | 38.870306 | 1.347972 | 60 |
|  |  | Punta des Trencs | Tre | 38.969194 | 1.270722 | 68 |
|  |  | Punta sa Pedrera | Ped | 38.970028 | 1.261111 | 101 |
|  |  | Ses Salines | Sal | 38.746806 | 1.432889 | 100 |
|  |  | Platja ses Canyes | Can | 38.729528 | 1.451861 | 103 |
|  |  | Platja Migjorn1 | Mig1 | 38.684389 | 1.467500 | 100 |
|  |  | Platja Migjorn2 | Mig2 | 38.680806 | 1.481972 | 100 |
| *S. psammitis* | Spain | Jatar | Jat | 36.916194 | -3.905028 | 82 |
|  |  | Benahavís | Ben | 36.511000 | -5.035750 | 92 |
|  |  | Ojén | Oje | 36.592972 | -4.857389 | 100 |
|  |  | Sierra de Gredos | Gre | 40.215608 | -5.247733 | 100 |
