# Supplementary material for: Diversity of sexual systems within different lineages of the genus Silene
Source: AoB Plants. 2015 May 15;7:plv037. doi: 10.1093/aobpla/plv037 (PMC4433491; doi:10.1093/aobpla/plv037)
Supplement: Additional Information [file supp_plv037_plv037supp_file2.docx]

**File 2**. Revised literature that were not cited in the manuscript because no information about the sexual system of species was found.

Davis PH, Mill RR, Tan K. 1988. *Silene* L. In: Davis PH, Mill RR Tan K, eds. *Flora of Turkey and the East Aegean Islands*, vol. 10. Edinburgh: Edinburgh University Press.

Pignatti S. 2003. Flora d’Italia, 3rd edn. Edagricole

Fielding, J, Turland N. 2008. *Flowers of Crete*. Kew: Royal Botanic Gardens.

Goldblatt P, Manning J. 2000. *Cape Plants. A conspectus of the Cape flora of South Africa*. Streliztia 9.

Meikle RD. 1977. *Flora of Cyprus*. Kew: Royal Botanic Gardens.

Ledebour CF Von. 1842. *Flora Rossica*, vol. 1. Stuttgart: Schweizerbart.

Lauber K, Wagner G. 2007. *Flora Helvetica*. Paris: Belin

Stace C. 1997. *New Flora of the Bristish Isles*, 2nd edn. Cambridge: Cambridge University Press.

Jeanmonod D, Gamisans J. 2007. *Flora Corsica*. Édisud.

Sampaio G. 1988. *Flora Portuguesa*, 3rd edn. Instituto Nacional de Investigaçao Cientifica.

eFloras. 2008. Published on the Internet <http://www.efloras.org> [accessed November 2014] Cambridge, MA: Missouri Botanical Garden, St. Louis, MO & Harvard University Herbaria.
