# Supplementary material for: Diversity of sexual systems within different lineages of the genus Silene
Source: AoB Plants. 2015 May 15;7:plv037. doi: 10.1093/aobpla/plv037 (PMC4433491; doi:10.1093/aobpla/plv037)
Supplement: Additional Information [file supp_plv037_plv037supp_file3.docx]

|  |  |  | **Number of flowers per morph** | | | | | | % FF in | |
| --- | --- | --- | --- | --- | --- | --- | --- | --- | --- | --- |
|  |  | % FF per | **Females** | | **Hermaphrodites** | | **Gynomonoecious** | | GM plants | |
| SPECIES | POP | population | Mean (N) | SE | Mean (N) | SE | Mean (N) | SE | Mean | SE |
|  | Roq | 10.4 | 1.7 (13) | 0.4 | 4.4 (76) | 0.7 | 7.7 (9) | 2.3 | 32.0 | 6.3 |
|  | Gat | 1.3 | 1.0 (2) | 0.0 | 3.0 (98) | 0.3 | 5.5 (2) | 1.5 | 19.6 | 5.4 |
|  | Odi | 5.9 | 4.0 (1) | - | 5.3 (82) | 0.4 | 4.7 (18) | 0.7 | 37.3 | 3.9 |
|  | Vic | 2.1 | 1.2 (5) | 0.2 | 3.0 (95) | 0.4 | - | - | - | - |
| *S littorea* | Alj | 2.2 | 3.7 (6) | 1.3 | 12.0 (93) | 1.6 | 7.0 (1) | - | 42.9 | - |
|  | Cas | 10.6 | 1.8 (9) | 0.7 | 2.0 (86) | 0.2 | 3.4 (5) | 0.8 | 41.7 | 9.1 |
|  | Agu | 4.2 | 1.8 (4) | 0.5 | 2.9 (92) | 0.2 | 5.0 (4) | 0.7 | 26.7 | - |
|  | Man | 18.7 | 1.8 (18) | 0.3 | 3.0 (64) | 0.3 | 5.1 (18) | 1.2 | 36.4 | - |
|  | Car | 12.9 | 2.3 (11) | 0.5 | 2.3 (83) | 0.2 | 4.0 (6) | 0.9 | 30.4 | 5.3 |
| Mean ± s.e. | | **7.6 ± 2.0** | **2.0** | **0.2** | **4.3** | **0.3** | **5.14** | **0.5** | **34.8** | **2.0** |
|  | Ger | 3.2 | 1.0 (4) | 0.0 | 1.6 (94) | 0.1 | 2.0 (1) | - | 50.0 | - |
|  | Tab1 | 7.1 | 1.0 (1) | - | 1.3 (99) | 0.1 | - | - | - | - |
| *S. adscendens* | Tab2 | 0.8 | 2.4 (11) | 0.8 | 4.2 (87) | 0.3 | 2.0 (2) | 0.0 | 50.0 | 0.0 |
|  | Feo | 2.9 | 1.3 (3) | 0.3 | 2.0 (95) | 0.1 | 5.5 (2) | 2.5 | 22.9 | 10.4 |
|  | Tor | 10.2 | 1.3 (6) | 0.2 | 1.4 (62) | 0.1 | 4.0 (4) | 0.7 | 28.8 | 7.2 |
| Mean ± s.e. | | **4.8 ± 1.7** | **1.7** | **0.4** | **2.1** | **0.1** | **3.7** | **0.7** | **34.5** | **5.2** |
|  | Tre | 1.1 | 1.0 (1) | - | 1.3 (67) | 0.1 | - | - | - | - |
|  | Ped | 2.3 | 1.5 (2) | 0.5 | 2.1 (97) | 0.2 | 3.5 (2) | 0.5 | 29.2 | 4.2 |
|  | Sal | 9.8 | 1.3 (9) | 0.2 | 1.5 (88) | 0.1 | 2.3 (3) | 0.3 | 44.4 | 5.6 |
|  | Can | 8.0 | 1.1 (8) | 0.1 | 1.1 (94) | 0.0 | 2.0 (1) | - | 50.0 | - |
| *S. cambessedesii* | Mig1 | 14.1 | 1.9 (15) | 0.5 | 3.0 (65) | 0.4 | 14.1 (20) | 3.7 | 26.6 | 3.7 |
|  | Mig2 | 10.2 | 1.0 (11) | 0.0 | 1.2 (88) | 0.1 | 2.0 (1) | - | 50.0 | - |
|  | Cav | 11.1 | 1.0 (13) | 0.0 | 1.4 (84) | 0.1 | 3.3 (3) | 1.3 | 38.9 | 11.1 |
|  | Mos | 3.6 | 1.0 (3) | 0.0 | 1.4 (57) | 0.1 | - | - | - | - |
| Mean ± s.e. | | **7.5 ± 1.7** | **1.3** | **0.1** | **1.6** | **0.1** | **10.3** | **2.7** | **31.4** | **3.0** |
|  | Jat | 18.6 | 2.5 (12) | 0.6 | 2.2 (67) | 0.2 | 3.0 (3) | 0.6 | 44.4 | 5.6 |
|  | Ben | 7.1 | 1.0 (2) | 0.0 | 2.4 (80) | 0.2 | 4.7 (10) | 0.8 | 36.6 | 5.0 |
| *S. psammitis* | Oje | 29.4 | 2.2 (18) | 0.3 | 1.9 (66) | 0.1 | 3.3 (16) | 0.4 | 47.5 | 4.4 |
|  | Gre | 9.5 | 2.4 (8) | 0.5 | 2.0 (92) | 0.1 | - | - | - | - |
| Mean ± s.e. | | **16.1 ± 5.1** | **2.3** | **0.3** | **2.1** | **0.1** | **3.8** | **0.4** | **43.4** | **3.1** |

**File 3**. Percentage of female flowers (FF) in populations, average number of flowers per sexual morph and percentage of female flowers in gynomonoecious (GM) plants. Mean ± s.e. per species is highlighted in bold.
